# Integrative Analysis of Human Macrophage Inflammatory Response Related to *Mycobacterium tuberculosis* Virulence

**DOI:** 10.3389/fimmu.2021.668060

**Published:** 2021-06-28

**Authors:** Pauline Bade, Fabrizio Simonetti, Stephanie Sans, Patricia Laboudie, Khadija Kissane, Nicolas Chappat, Sophie Lagrange, Florence Apparailly, Christine Roubert, Isabelle Duroux-Richard

**Affiliations:** ^1^ Institute for Regenerative Medicine & Biotherapy (IRMB), INSERM, Univ Montpellier, CHU Montpellier, Montpellier, France; ^2^ Evotec ID (Lyon), Lyon, France

**Keywords:** miRNA, *Mycobacterium tuberculosis*, *Mycobacterium marinum*, macrophage, host response, inflammatory response, virulence

## Abstract

*Mycobacterium tuberculosis* (Mtb), the etiological agent of tuberculosis, kills 1.5 to 1.7 million people every year. Macrophages are Mtb’s main host cells and their inflammatory response is an essential component of the host defense against Mtb. However, Mtb is able to circumvent the macrophages’ defenses by triggering an inappropriate inflammatory response. The ability of Mtb to hinder phagolysosome maturation and acidification, and to escape the phagosome into the cytosol, is closely linked to its virulence. The modulation of the host inflammatory response relies on Mtb virulence factors, but remains poorly studied. Understanding macrophage interactions with Mtb is crucial to develop strategies to control tuberculosis. The present study aims to determine the inflammatory response transcriptome and miRNome of human macrophages infected with the virulent H37Rv Mtb strain, to identify macrophage genetic networks specifically modulated by Mtb virulence. Using human macrophages infected with two different live strains of mycobacteria (live or heat-inactivated Mtb H37Rv and *M. marinum*), we quantified and analyzed 184 inflammatory mRNAs and 765 micro(mi)RNAs. Transcripts and miRNAs differently modulated by H37Rv in comparison with the two other conditions were analyzed using in silico approaches. We identified 30 host inflammatory response genes and 37 miRNAs specific for H37Rv virulence, and highlight evidence suggesting that Mtb intracellular-linked virulence depends on the inhibition of IL-1β-dependent pro-inflammatory response, the repression of apoptosis and the delay of the recruitment and activation of adaptive immune cells. Our findings provide new potential targets for the development of macrophage-based therapeutic strategies against TB.

## Introduction

Tuberculosis is one of the top 10 causes of death (1) and, right after COVID-19 (2), is the world’s deadliest infection caused by a single infectious agent: *Mycobacterium tuberculosis* (Mtb) (3). Mtb is responsible for 1.5 to 1.7 million deaths each year, but unlike COVID-19, is more prevalent in low and middle income countries (4). Active tuberculosis infection (ATBI) is characterized by replicating and metabolically active bacteria and causes severe lung lesions while latent tuberculosis infection (LTBI) remains asymptomatic. Seventy percent of newly infected individuals efficiently clear the bacteria. However, for the remaining 30%, the immune system is only able to contain, but not to eradicate the bacteria (5). These people develop LTBI that can persist for their entire lifetime (6). However, approximately 5 to 10% of LTBIs evolve into ATBIs when the immune system is no longer able to constrain Mtb (5). The ability of Mtb to persist for so long in the host is due to its ability to hijack host defense mechanisms by converting macrophages into a permissive cellular niche (7, 8).

Macrophages are first-line antimicrobial cells in our body. They play a key role during Mtb infection by triggering an arsenal of immune responses (7). Macrophages are the most abundant cell type within the site of infection and represent the primary host cells for Mtb (9). Depending on the polarization of their immune response, macrophages ability to fight Mtb differs. Classically activated (M1-like) macrophages trigger a Th1 pro-inflammatory response and help to eliminate the bacteria. Alternatively, activated (M2-like) macrophages trigger a Th2 anti-inflammatory response and become long term hosts (10–13). Many studies describe a strong pro-inflammatory response promoting M1-like polarization, immediately after Mtb infection, followed later by a shift to an anti-inflammatory response and M2-like polarization (14–17). However, pro-inflammatory mechanisms do not always kill the bacteria and anti-inflammatory mechanisms are not always protective for the host. Inability to orchestrate a protective inflammatory response leads to poor clinical outcome (9). This might be the reason why innate immunity is often related to genetic susceptibility to tuberculosis (18–20) and why cytokine signaling and inflammation pathways are found to be the most induced processes during the infection (21, 22). MicroRNAs (miRNAs) are critical regulators of the fine-tuning of cytokine signaling, macrophage polarization, and inflammation. They are small endogenous non-coding RNA molecules, acting mainly as post-transcriptional repressors by targeting mRNAs on their 3’UTR (23–27). Several studies have explored the roles of host miRNAs in tuberculosis and showed that Mtb infection modifies the miRNome of macrophages (28–31). Mtb might thus influence the macrophage inflammatory response by modulating their miRNAs expression.

Mtb encompasses a group of genetically related species (named the Mtb complex) that are highly contagious airborne mycobacterial strains and can cause tuberculosis in humans (32). *Mycobacterium* (*M.) marinum* is one of the most closely related mycobacterium species to the Mtb complex and a commonly used pertinent intracellular infection model. Indeed, both thrive in the same macrophage compartments (33) and their survival in the immature phagosome is made possible through pathogen-dependent inhibition of phagosome-lysosome fusion. Then, both Mtb and *M. marinum* use the ESX-1 secretion system to escape the phagosome into the cytosol and thus to circumvent macrophage defenses (34–39). Cutaneous lesions caused either by Mtb or *M. marinum* form similar granulomas in humans (40), suggesting that they trigger comparable immune responses. The *M. marinum* ESX-1 system however confers reduced virulence, compared to that of Mtb (41), and human granulomatous lesions are less severe than those caused by Mtb infection (42). This suggests that phagosome escape is not the only cause of mycobacterial virulence. After phagosomal escape, Mtb must be able to reprogram macrophages’ defenses in a way that favors its virulence in humans in a more effective way than *M. marinum*.

Recent studies have indicated that genetic diversity within the Mtb complex can influence host inflammatory response to infection and during tuberculosis disease (43). Because Mtb strains that diminish protective cytokine secretion show enhanced virulence (44, 45), the present study aims at identifying how the virulent Mtb strain H37Rv specifically reprograms the inflammatory transcriptome and related miRNAs of macrophages in comparison with less virulent Mtb strains. compared with *M. marinum*. We thus compared macrophages’ inflammatory transcriptomes following infection with either the virulent Mtb strain H37Rv, *M. marinum* or the heat killed avirulent H37Ra strain (HKMT). *M. marinum* infection was used to filter the response specific for H37Rv with a less, yet virulent mycobacterial strain, which is also able to escape the phagosome and to trigger a cytosolic immune response. We used HKMT infection as a way to focus on both post-phagocytosic changes and to identify changes related to human-specific pathogen recognition. Indeed, in contrast to H37Rv, macrophages successfully eliminate HKMT in their phagolysosomes (46). As a macrophage model, we used PMA-differentiated THP-1 cells since they display similar properties compared to human monocyte-derived macrophages during Mtb infection (47). We identified an H37Rv-specific signature of inflammatory genes. We also identified a miRNA-based signature following H37Rv infection and provide potential mRNA/miRNA circuits related to H37Rv virulence in the context of the macrophage inflammatory response.

## Materials and Methods

### Mycobacterial Strains and Cultures

We used fluorescent H37Rv and *M. marinum* strains that were both obtained as a kind gift from IPBS, Toulouse. H37Rv and *M. marinum* cultures were grown at 37°C or 33°C, respectively, and 5% CO2 in Middlebrook 7H9 broth supplemented with 10% Middlebrook Oleic Albumin Dextrose Catalase (Difco, Livonia, MI). Cultures were grown to a mid-log phase (optical density 600 [OD600] of 0.6), then frozen with 10% glycerol at −80°C in 1.5 ml aliquots prior to infection. Heat inactivated H37Ra (HKMT) was purchased from Invivogen.

### Cell Cultures

The human monocytic cell line THP-1 cells (TIB-202) were purchased from ATCC. Cells were cultured in 24 well plates (Corning) and differentiated into THP-1 derived macrophages 24 hours prior to infection with 40 ng/mL phorbol 12-myristate 13-acetate (PMA) in RPMI 1640 Medium GlutaMAX™ Supplement (Gibco) at 37°C and 5% CO2. Experiments were realized within 15 passages and cell viability was measured before experiments by trypan blue exclusion and was greater than 97%. CD14^+^ primary human monocytes were extracted from peripheral blood of 5 healthy donors anonymously provided by the French Blood Establishment (EFS, Lyon). CD14^+^ monocytes were purified from whole blood using an autoMACS^®^ Pro Separator, Whole Blood Column Kit and StraightFrom^®^ Whole Blood CD14 MicroBeads (Mitenyi) according to the manufacturer’s instructions. Cell viability was measured before proceeding to macrophage differentiation, by trypan blue exclusion and was always greater than 80%. CD14^+^ monocytes were then plated in 24 well plates (Corning) at a density of 400 000 cells per well and differentiated into macrophages for 5 days prior to infection with 50 µg/ml Rh-GMCSF (Miltenyi) in RPMI 1640 Medium GlutaMAX™ Supplement (Gibco) with 10% FBS (Sigma-Aldrich) at 37°C, 5% CO2.

### Infections

Macrophages were infected with H37Rv or *M. marinum* at a multiplicity of infection (MOI) of 1:1 for 1 hour and at an MOI of 4: 1 for 3 hours, respectively, in RPMI 1640 medium GlutaMAX™ Supplement with 10% HiFBS. Macrophages stimulation with HKMT was performed with 50 µg/ml of HKMT for 3 hours in RPMI 1640 medium GlutaMAX™ Supplement with 10% HiFBS. Cells were then rinsed with phosphate buffered saline (PBS) to remove extracellular mycobacteria and further cultured in RPMI 1640 medium GlutaMAX™ Supplement with 10% HiFBS for 48 hours before RNA extraction. Uninfected cells were handled in the same conditions as infected cells and served as controls.

### Gene and miRNA Profiling

Total RNA including miRNAs was extracted 2 days post-infection. Briefly, to protect RNA from degradation, macrophages were rinsed with PBS, then scraped with Maxwell^®^ RSC miRNA Tissue Kit homogenization solution/thioglycerol (50/1) (Promega). Followed 10 min of incubation with Maxwell^®^ RSC miRNA Tissue Kit lysis buffer (Promega), cells and bacteria were lysed by bead beating into Matrix B tubes containing silica beads (MP Biomedical) with the Super-Fast Prep-1 instrument (MP Biomedical). Finally, samples were processed into a Maxwell^®^ RSC instrument for RNA extraction. RNA concentration was measured with QIAxpert System (Qiagen) and RNA integrity was evaluated by automated electrophoresis with TapeStation Systems (Agilent). Reverse transcription of total mRNA or miRNAs were performed with 500 ng total RNA using SuperScript™ IV VILO™ Master Mix or a Taqman™ microRNA Reverse Transcription kit (Applied Biosystems), respectively. qPCR amplifications were run with a QuantStudio™ 12K Flex system (Applied Biosystems) and using a customized TaqMan^®^ Array (**Table S1** in **Supplementary Material**) for mRNAs and the TaqMan^®^ Array Human MicroRNA Card Set v3.0 dispatched in two pools: highly characterized miRNAs (pool A) and more recently discovered miRNAs (pool B) (Applied Biosystems) for miRNAs, according to the manufacturer’s instructions.

### Data and Statistical Analysis

The data was analyzed using the ThermoFisher Connect™ online application (ThermoFisher). The mRNA content relative to the secreted proteins was normalized to GAPDH and GUSB expression, that of the receptors was normalized to GAPDH and TBP expressions and miRNA content was normalized using the global normalization method. Then, relative expression and expression fold changes were calculated following the ΔCt and 2^ΔΔCt^ methods, respectively. Briefly: ΔCt = Ct_gene_ – Ct_endogenous control_ and ΔΔCt = ΔCt treated sample – ΔCt untreated control. Heatmaps and principal components analysis (PCA) were generated with Clustvis online software (48). All miRNA and gene Taqman Low Density Array data are available from the ncbi database: https://www.ncbi.nlm.nih.gov/geo/query/acc.cgi?acc=GSE165327. Genes and miRNAs interactions were further explored with the Ingenuity Systems™ Pathways Analysis (IPA) tool (http://www.ingenuity.com). We used GraphPad Prism 8 for statistical analysis. Student’s t-test was used on ΔCt to compare treated samples to their controls and on ΔΔCt to compare H37Rv-infected PMA differentiated THP-1 to H37Rv-infected CD14^+^ (monocyte-derived macrophages) MDM responses. ANOVA with multiple comparisons were used on ΔΔCt to compare H37Rv-infected macrophages to *M. marinum*-infected macrophages and to HKMT-stimulated macrophages. PCA and hierarchical or unsupervised clustering were performed using Clustvis online software with the correlation distance method and average linkage. Relative gene expression or gene expression fold changes as indicated were centered and unit variance scaled. Imputation was used for missing value estimation.

## Results

### Infection With Virulent *Mycobacterium tuberculosis* Leads to a Specific Deregulation of Macrophage Host Response-Related Genes

**Figure 1:**
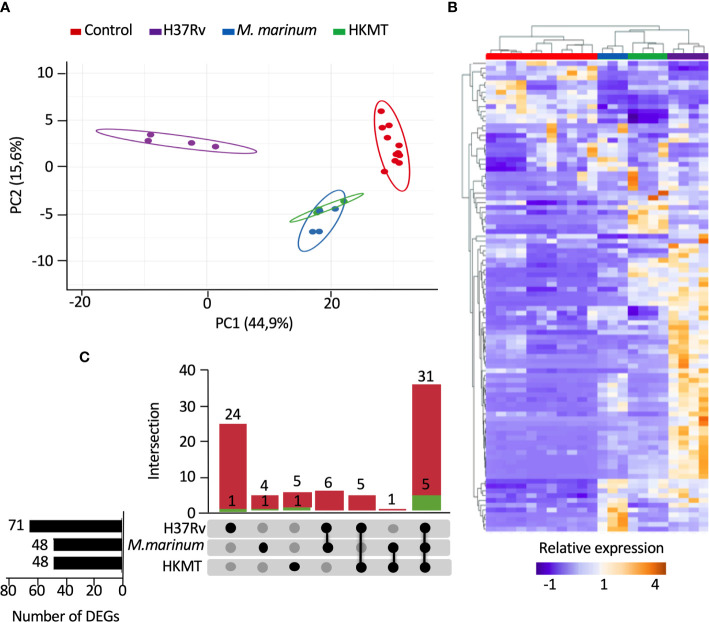
Infection with H37Rv led to a specific deregulation of macrophage inflammatory response-related genes. Macrophages were infected with H37Rv, *M. Marinum* or stimulated with HKMT for 48 hours, then total RNA was extracted and the expression levels of 184 host immune response-related genes were quantified using RT-PCR (n = 11 control, n = 3 *M. Marinum*, n=4 HKMT and H37Rv). For each experiment with the different mycobacterial strains, uninfected macrophages were used as control. Using Clustvis software, hierarchical clustering and principal component analysis were generated for genes, which after PCR had a CT value below 32 (98 genes). **(A)** Unsupervised PCA representation of the samples distribution depending on their overall relative gene expression following infection with H37Rv, *M. marinum* and stimulation with HKMT. Imputation was used for missing value estimation. **(B)** UpSet diagram representing the intersection of the DEGs relative to H37Rv or *M. marinum* infections or HKMT as compared with uninfected controls. In green: downregulated genes count, in red: upregulated genes count. **(C)** Unsupervised hierarchical clustering of the samples distribution depending on the overall relative expression of 93 genes following infection with H37Rv, *M. marinum* or stimulation with HKMT. Imputation was used for missing value estimation and samples were clustered using the correlation distance method and average linkage.

To compare the host inflammatory response induced by macrophages upon infection with a virulent or non-virulent mycobacterial strain, we used PMA-differentiated THP-1 cells as a human macrophage model and infected them with either the virulent H37Rv strain, or the occasional human pathogen *M. marinum* and the avirulent heat killed Mtb (HKMT). In this first attempt to dissect the host response associated with specific Mtb virulence, we decided to use the classical laboratory strain H37Rv as a surrogate for Mtb strains. Using RT-qPCR arrays, the host inflammatory response was evaluated by quantifying the expression level of 184 genes designed to encompass the full landscape of host immune responses (**Table S1** in **Supplementary Material**). Among the 184 mRNAs tested, 98 genes were detectable (Ct values < 32) in THP-1, including 14 interleukins, 11 interleukin receptors, 23 chemokines, 12 chemokine receptors, 6 interferons, 2 interferon receptors, 11 tumor necrosis factor (TNF) superfamily members, 12 TNF receptors and 7 toll-like receptors (**Table S2** in **Supplementary Material**). We used principal component analysis (PCA) and unsupervised hierarchical clustering to visualize the relationships between the three sample groups, based on the expression of these genes. PCA analysis displayed 60.5% of the dataset on the two principal components (**Figure 1A**), which was representative of the sample distribution. Interestingly, PCA showed that expression profiles obtained with *M. marinum* infection and HKMT stimulation were similar, as their distribution overlapped, and that both were very different from H37Rv infection. This was confirmed by the unsupervised hierarchical clustering analysis (**Figure 1B**). H37Rv-infected samples were found further from controls compared to the other two conditions on the PCA and heatmap (**Figures 1A, B**), showing that infection with H37Rv induced more drastic changes than the other two experimental conditions.

First, we assessed which genes are differentially expressed between uninfected and infected conditions. The UpSet diagram (49) represents the intersection between the three sets of differentially expressed genes (DEGs) from the three infections (**Figure 1C**). The data indicate that H37Rv infection significantly deregulated 71 DEGs, whereas both other conditions only modulated 48 DEGs. While 36 DEGs were shared between the three conditions, 25 DEGs were specific for H37Rv infection and only 5 and 6 DEGs for *M. marinum* and HKMT, respectively. Overall, our data showed that H37Rv infection induced greater changes to the host response, with more DEGs and higher fold changes.

**Figure 2:**
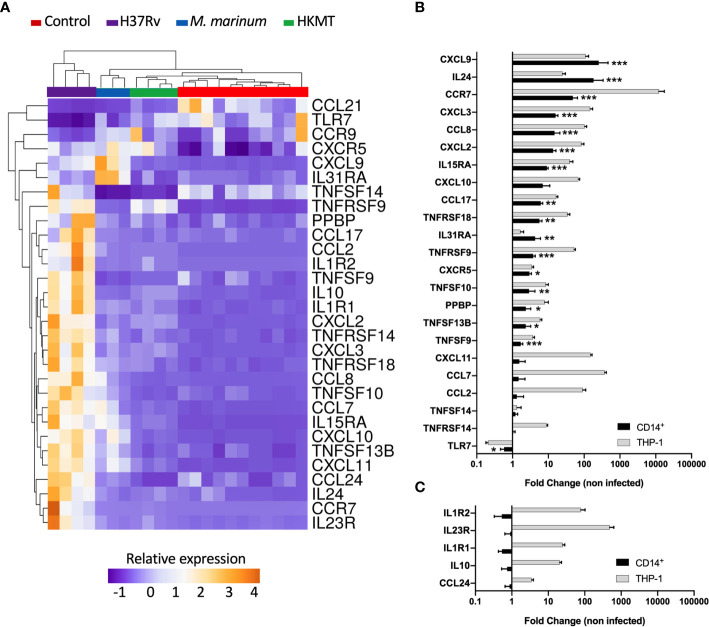
Infection with H37Rv led to a specific and significantly different host inflammatory response. **(A)** THP-1 differentiated into macrophages were infected with H37Rv, *M. Marinum* or stimulated with HKMT for 48 hours, then total RNA was extracted and the expression level of the 184 host immune response-related genes were quantified using RT-PCR (n = 11 control, n = 3 *M.marinum*, n=4 HKMT and H37Rv). For each experiment with the different mycobacterial strains, uninfected macrophages were used as control, independently. Using Clustvis software, a supervised hierarchical clustering was generated for the 98 genes with CT value below 32. Supervised hierarchical clustering of macrophage samples distribution depending on the relative expression of genes that were significantly deregulated by H37Rv infection as compared with both *M. marinum* infection and HKMT stimulation. Statistical analysis was performed on ΔΔCt using ANOVA and multiple comparisons. Imputation was used for missing value estimation. Samples were clustered using the correlation distance method and average linkage. **(B, C)** THP-1 and CD14^+^ MDM were infected with H37Rv for 48 hours, then total RNA was extracted and the expression level of the 30 H37Rv-specific host inflammatory response was quantified using RT-PCR (n = 4). Graphs represent gene expression fold change compared to uninfected cells. Statistical analysis was performed on ΔΔCt using Student’s t-test. *p value < 0.05, **p value < 0.01, ***p value < 0.001.

Second, to identify how macrophages specifically respond to H37Rv, we compared the expression levels of all DEGs from the three infections (**Table 1** and **Figure 2A**). For each DEG, we considered that the expression was specific for H37Rv infection if the fold change versus *M. marinum* or HKMT was greater than or equal to 2 and p-value < 0.05. Among the 25 DEGs specific for H37Rv, six genes [*CCL17*, *CCL24*, *PPBP* (*CXCL7*), *CXCR5*, *CCR9* and *TLR7*] were significantly deregulated following H37Rv infection only and not in the other two conditions. On the other hand, *IL24*, *IL31RA* and *TNFSF14* were not significantly deregulated by H37Rv, whereas they were deregulated by HKMT, *M. marinum*, and both, respectively (**Table S2** in **Supplementary Material**).

**Table 1:** H37Rv specific host response gene set.

Gene	Gene description	H37Rv/M.marinum		H37Rv/Hi-H37Ra	
		Fold change	P-value	Fold change	P-value
**Genes upregulated by H37Rv as compared with *M. marinum* and HKMT**					
IL23R	interleukin 23 receptor	49.44	<0.001	123.00	<0.001
CCR7	chemokine (C-C motif) receptor 7	48.62	0.001	115.06	<0.001
IL1R1	interleukin 1 receptor, type I	8.77	<0.001	8.51	<0.001
TNFSF14^(e)^	tumor necrosis factor superfamily member 14	8.21	0.001	5.11	0.002
TNFRSF9	tumor necrosis factor receptor superfamily, member 9	8.19	0.001	3.32	<0.001
TNFRSF18	tumor necrosis factor receptor superfamily, member 18	7.22	0.001	6.81	0.001
CCL17^(a)^	C-C motif chemokine ligand 17	6.79	0.027	7.52	0.016
PPBP^(a)^	pro-platelet basic protein	5.19	0.013	6.11	0.005
IL1R2	interleukin 1 receptor, type II	4.87	0.017	6.87	0.004
CCR9^(a)^	chemokine (C-C motif) receptor 9	4.69	0.047	6.52	0.014
CCL7	C-C motif chemokine ligand 7	4.63	0.013	45.49	<0.001
IL24^(c)^	interleukin 24	4.41	0.005	4.77	0.002
TNFSF9^(b)^	tumor necrosis factor superfamily member 9	3.99	0.001	3.40	0.002
IL10	interleukin 10	3.94	<0.001	2.65	0.000
TNFRSF14	tumor necrosis factor receptor superfamily, member 14	3.75	0.001	4.69	<0.001
CXCL2	C-X-C motif chemokine ligand 2	3.51	0.005	3.42	0.004
CXCL3	C-X-C motif chemokine ligand 3	3.51	0.004	3.43	0.002
CCL24^(a)^	C-C motif chemokine ligand 24	2.96	0.012	4.50	0.001
CCL2	C-C motif chemokine ligand 2	2.79	0.024	5.92	0.001
IL15RA	interleukin 15 receptor, alpha	2.34	0.032	7.33	<0.001
CXCR5^(a)^	chemokine (C-X-C motif) receptor 5	2.04	0.021	2.06	0.017
**Genes with intermediate expression: *M. marinum* H37Rv HKMT**					
CXCL11	C-X-C motif chemokine ligand 11	0.42	0.036	26.83	<0.001
CCL8	C-C motif chemokine ligand 8	0.09	0.002	13.57	0.001
CXCL9	C-X-C motif chemokine ligand 9	0.18	0.003	12.39	<0.001
TNFSF10	tumor necrosis factor superfamily member 10	0.36	0.050	12.33	<0.001
CXCL10	C-X-C motif chemokine ligand 10	0.15	0.001	10.11	<0.001
TNFSF13B	tumor necrosis factor superfamily member 13b	0.37	0.001	4.85	0.001
IL31RA^(d)^	interleukin 31 receptor, alpha	0.27	0.004	3.88	0.003
**Genes downregulated by H37Rv as compared with *M. marinum* and HKMT**					
TLR7^(a)^	toll-like receptor 7	0.26	0.004	0.31	0.009
CCL21	C-C motif chemokine ligand 21	0.06	0.001	0.06	0.001

^(a)^ Deregulated by H37Rv only; ^(b)^ deregulated by H37Rv and HKMT; ^(c)^ deregulated by HKMT only; ^(d)^ deregulated by M. marinum only; ^(e)^ deregulated by HKMT and M. marinum.

One hundred and eighty-four host immune response-related genes were quantified in RT-qPCR in THP-1 derived macrophages following two days infection with H37Rv (n=4), M. marinum (n=3), or two days stimulation with HKMT (n=4). Genes showing significantly different expression levels following H37Rv infection as compared with M. marinum infection and HKMT stimulation were identified using one-way ANOVA and multiple comparisons on ΔΔCt.

The majority of the genes that had an expression level specific for H37Rv infection belonged to the common DEGs induced by the three pathogens. Indeed, the H37Rv-specific host response led to a more drastic deregulation of 12 genes by either inducing upregulation of *CCR7, IL1R1, TNFRSF9, TNFRSF18, IL1R2, CCL7, IL10, TNFRSF14, CXCL2, CXCL3, CCL2* and *IL15RA* or inducing downregulation of *CCL21*. *CCR7* and *IL23R* both ranked as the first and second most upregulated genes, with fold changes equal to 19 and 115 for *CCR7*, and 50 and 123 for *IL23R*, respectively. *TLR7* and *CCL21* were the only two downregulated genes following H37Rv infection with respect to the uninfected controls. In addition, both genes were even more downregulated when comparing infection with H37Rv and with *M. marinum* or HKMT. *CCL21* ranked as the most downregulated gene with a 16-fold downregulation in H37Rv infection as compared with the other two conditions. H37Rv-infection of macrophages did not always lead to extreme expression profiles. Indeed, *CXCL11*, *TNFSF13B*, *TNFSF10*, *IL31RA*, *CXCL9*, *CXCL10*, *CCL8* expressions were intermediate, all downregulated as compared with *M. marinum* infection and upregulated as compared with HKMT stimulation. Overall, the H37Rv-specific host inflammatory response included 30 genes (**Figure 2A** and **Table 1**). Finally, we validated this finding in primary human macrophages. Using RT-qPCR, we quantified the expression level of the 30 H37Rv-specific genes in Mtb-infected macrophages differentiated from blood CD14^+^ monocytes of healthy donors. Results showed that expression levels were comparable between the human THP-1 macrophage cell line and primary macrophages for more than 80% of the genes (**Figure 2B**). Only 5 out of 30 genes did not show the same variation (**Figure 2C**). While *IL1R2*, *IL23R*, *IL1R1*, IL-*10*, and *CCL24* were highly upregulated upon infection of THP-1 macrophages, their expression was barely affected upon infection of primary macrophages.

### H37Rv Infection Leads to the Modulation of 37 miRNAs

**Figure 3:**
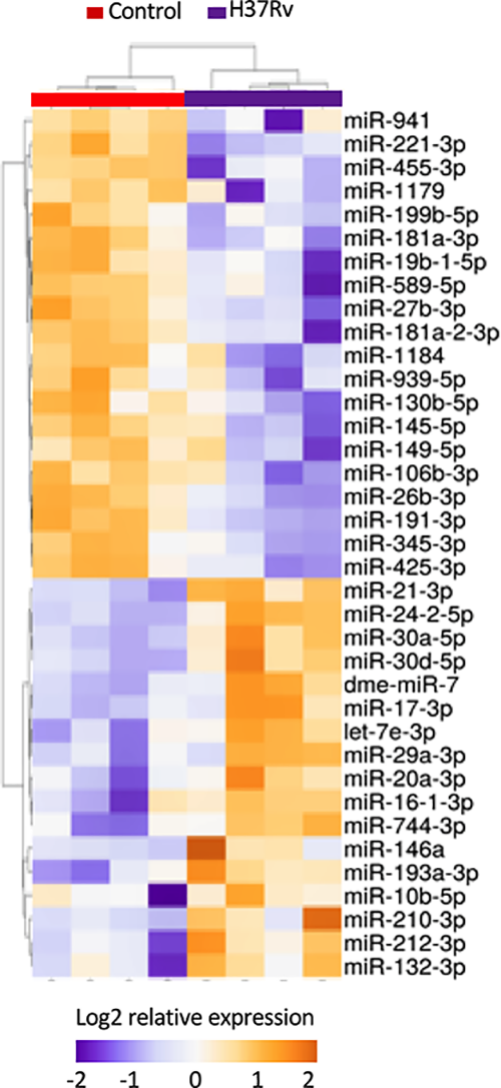
Expression levels of miRNAs significantly deregulated after H37Rv infection. THP-1 differentiated into macrophages were infected with H37Rv for 48 hours (n = 4). After total RNA including small RNA extraction, the expression level quantification of 762 human miRNAs was performed using TaqMan^®^ Array Human MicroRNA Card. Using Clustvis software, a supervised hierarchical analysis of the distribution of macrophage samples based on relative log2 expression of 37 miRNAs significantly modified by H37Rv infection compared to uninfected controls. Statistical analysis was performed on ΔCt using Student’s t test to identify the differently expressed miRNAs compared with uninfected controls. Imputation was used for missing value estimation. Samples were clustered using the correlation distance method and average linkage.

To further decipher virulence-mediated changes in the host response, we performed a global miRNA profiling of H37Rv-infected macrophages (**Figure 3**, **Table 2**). Of the 765 human miRNAs analyzed, up to 54.8% were detectable with a CT value < 32. When considering a fold change > 2 and a p-value < 0.05, 37 miRNAs were significantly deregulated following H37Rv infection as compared to the uninfected controls. Quantification of human miRNA expression levels showed that 17 miRNAs were upregulated and 20 were downregulated. As miRNAs belonging to the same family or cluster suggests that they co-regulate the same biological pathway, it seems relevant to highlight that, among the 37 differentially expressed miRNAs, there were two miRNA families: miR-30 family (miR-30a and miR-30b) and miR-181 family (miR-181a-1-3p and miR-181a-2-3p). We also observed that the miR-132/212 cluster was significantly increased. Using a miRNome published dataset of CD14^+^ isolated from the blood of TB patients (GSE70425), we compared the expression levels of the 37 H37Rv-related miRNAs between Mtb-infected THP-1 and primary CD14^+^. Results showed that 80% of miRNAs displayed comparable expression levels between both conditions (**Figure S1** in **Supplementary Material**).

**Table 2:** H37Rv mediated miRNA deregulation.

miRNA ID	H37Rv/Ctrl	
	Fold Change	P-value
dme-miR-7-5p	6.69	0.012
hsa-miR-21-3p	4.51	0.001
hsa-miR-146a-5p	3.37	0.004
hsa-miR-29a-5p	3.34	0.045
hsa-miR-17-3p	3.26	0.032
hsa-miR-212-3p	2.9	0.002
hsa-miR-10b-5p	2.86	0.018
hsa-miR-24-2-5p	2.72	0.001
hsa-miR-16-1-3p	2.66	0.026
hsa-miR-132-3p	2.44	0,010
hsa-miR-744-3p	2.2	0.024
hsa-miR-210-3p	2.15	0.015
hsa-miR-30a-5p	2.14	0.001
hsa-let-7e-3p	2.13	0.014
hsa-miR-193a-3p	2.11	0,010
hsa-miR-30d-5p	2.07	0.003
hsa-miR-20a-3p	2.01	0,020
hsa-miR-27b-3p	0.49	0.001
hsa-miR-345-5p	0.45	0.006
hsa-miR-19b-1-5p	0.45	0.014
hsa-miR-1179	0.44	0.022
hsa-miR-589-5p	0.43	0.018
hsa-miR-145-5p	0.38	0.001
hsa-miR-130b-5p	0.35	0.013
hsa-miR-181a-3p	0.35	0.004
hsa-miR-941	0.35	0.036
hsa-miR-106b-3p	0.35	0.013
hsa-miR-455-3p	0.34	0.002
hsa-miR-181a-2-3p	0.31	0.007
hsa-miR-26b-3p	0.29	0.001
hsa-miR-939-5p	0.28	0.048
hsa-miR-425-3p	0.25	0.005
hsa-miR-191-3p	0.24	0.001
hsa-miR-149-5p	0.24	0.011
hsa-miR-221-3p	0.23	0.001
hsa-miR-1184	0.23	0.036
hsa-miR-199b-5p	0.15	0.004

THP-1 derived macrophages were infected with H37Rv strain (n=4). Two days post-infection, total RNA including small RNA was extracted. Using TaqMan^®^ Array Human MicroRNA Cards, the quantification of 765 human miRNAs was performed simultaneously by RT-qPCR method. Statistical analysis was performed on ΔCt using Student’s t-test to identified DEGs as compared to the uninfected controls.

### mRNA/miRNA Inflammatory Pathways Specific for H37Rv

**Figure 4:**
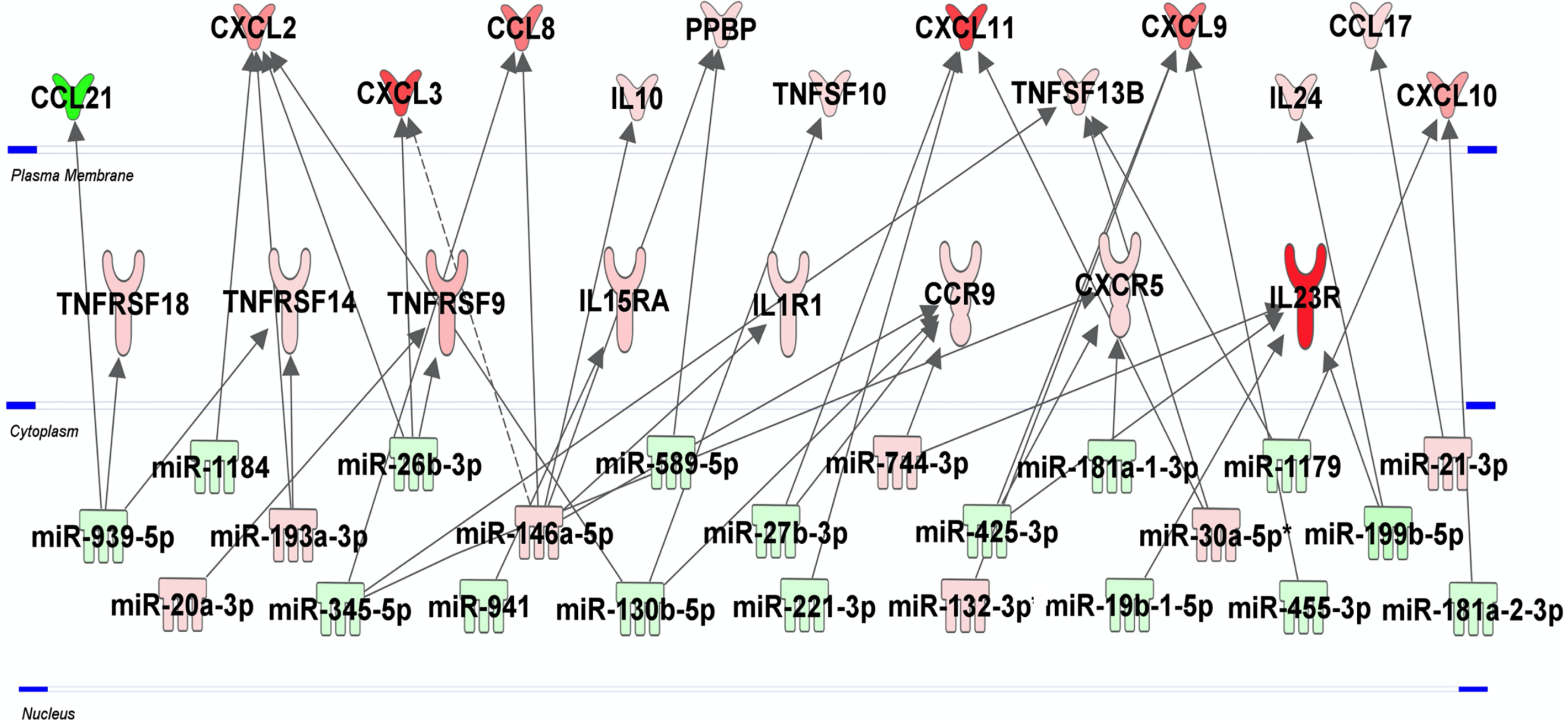
miRNA-mRNA networks generated with Ingenuity Pathway Analysis (IPA) for H37Rv-infected human macrophages. IPA analysis was performed to investigate the connection between the 30 and 37 H37Rv-specific host inflammatory response gene set and miRNAs, respectively. Filters were set to only include experimentally observed or high-confidence-predicted miRNA-mRNA interaction partners. Genes and miRNAs were colored in red or green depending on whether they were upregulated or downregulated (respectively) with respect to their uninfected controls and the shading intensity shows to what degree each miRNA or gene was deregulated. Direct and indirect miRNA-gene interactions are represented with a solid and dotted lines, respectively.

To identify predicted target genes and related pathways and to provide experimentally verified information on miRNA-target interactions, we performed an automated text-mining search analysis. The thirty seven miRNAs identified as dysregulated in H37Rv-infected macrophages were examined using miRWalk database (50). We found between 11 and 699 putative target genes for each of the 37 miRNAs. We applied a filter consisting in identifying genes targeted by at least 3 miRNAs and found 320 genes (**Table S3** in **Supplementary Material**). Using the Enrich database (51), gene ontology analysis revealed significant pathways with high Enrich combined score level such as cell cycle, FoxO signaling and P53 pathway with adjusted p-values 3.710^-7^, 1.110^-5^ and 6.210^-5^, respectively (**Table S4** in **Supplementary Material**). Interestingly, we found that 28 genes were targeted by at least five miRNAs (**Table S3** in grey color) and almost 70% of them have already been described as involved in Mtb infection (**Table S3** in **Supplementary Material**). The *HSPA1B* gene, also called *HSP72*, is targeted by 9 miRNAs, and the two genes *LDLR* and *NUFIP2* are targeted by 7 miRNAs. The integrative analysis of the 30 and 37 H37Rv-specific host inflammatory response genes and miRNAs, respectively, has been carried out to find possible relationships. In this attempt, we used the Ingenuity Pathway Analysis software program, associated with a miRNA target filter analysis, to identify miRNA-target genes and mRNA-mRNA interactions (**Figure 4**). miR-939-5p, miR-1184, miR-20a-3p, miR-193a-3p, miR-345-p, miR-26b-3p, miR146a-5p, miR-941, miR589-5p, miR-130b-5p, miR-27b-3p, miR-221-3p, miR-744-3p, miR-425-3p, miR-132-3p, miR-181a-1-3p, miR-181a-2-3p, miR-19b-1-5p, miR-30a-5p, miR-1179, miR-455-3p, miR-199b-5p and miR-21-3p, all targeted at least one inflammatory response genes related to Mtb virulence. Together, these 23 miRNAs might regulate 21 of the 34 H37Rv-specific genes: *CCL21*, *CXCL2*, *CXCL3*, *CCL8*, *IL10*, *PPBP*, *TNFSF10*, *CXCL11*, *TNFSF13B*, *CXCL9*, *IL24*, *CCL17*, *CXCL10*, *TNFRSF18*, *TNFRSF14*, *TNFRSF9*, *IL15RA*, *IL1R1*, *CCR9*, *CXCR5* and *IL23R*. Some miRNAs targeted more than 3 genes and thus might be more involved in the H37Rv-specific host inflammatory response than others. MiR-939-5p, miR-345-5p, miR-130b-5p, miR-425-5p, miR-26b-3p targeted 3 to 4 H37Rv-specific host inflammatory response genes and miR-146a targeted 6 genes. The most targeted H37Rv-specific host inflammatory response genes were *CXCL2*, *CXCL11*, *CCR9*, *TNFSF13B*, *CXCL9* and *IL23R*. miR-939-5p, miR-1184, miR-345-5p, miR-26b-3p, miR-941, miR-589-5p, miR-130-5p, miR-27b-3p, miR-221-3p, miR-425-3p, miR-181a-1-3p, miR-19b-1-5p, miR-1179, miR-455-3p, miR-119b-5p and miR-181a-2-3p targeted *TNFRSF18/TNFRSF14*, *CXCL2*, *CCL8/TNFSF13B/CXCR5*, *CXCL2/CXCL3/TNFRSF9*, *IL15RA*, *PPBP*, *CXCL2/TNFSF10/CCR9*, *CXCL11/CCR9*, *CXCL11*, *CXCL9/CXCR5/IL23R*, *CXCR5*, *IL23R*, *TNFSF13B/CXCL10*, *CXCL9*, *IL23R/IL24* and *CXCL10* with negative correlations (i.e. downregulated miRNAs correlated to upregulated genes), respectively. Altogether, 61% of the H37Rv-specific host inflammatory response gene set was targeted by 62% of the H37Rv deregulated miRNAs.

## Discussion

Macrophages are the primary host for Mtb and their inflammatory response is critical for the infection outcome (9). Although Mtb and *M. marinum* can both circumvent the phagolysosome fusion and escape to the cytosol, Mtb remains more virulent than *M. marinum* in humans. We hypothesized that Mtb virulence is enhanced by its capacity to alter the host inflammatory response. We sought to identify H37Rv-specific mRNA and miRNA genes, to identify pathways of the macrophage inflammatory response that might be related to Mtb virulence. As a model for human macrophages, we used the PMA-differentiated THP-1 cell line (47) to compare the transcriptomic changes induced in the inflammatory response by either H37Rv, *M. marinum* or HKMT. We identified 30 genes and 37 miRNAs that were differently expressed upon H37Rv infection and studied their relationships and associated cellular signaling pathways. Importantly, at least 80% of the H37Rv-specific genes and miRNAs identified with the THP1 model are confirmed in primary macrophage cells, validating our experimental approach.

Our unbiased analysis of the H37Rv–related miRNAs with their target genes experimentally validated, revealed their involvement in Mtb infection. HSP72 and LDLR were the most targeted genes by the H37Rv-specific miRNAs. These genes are involved in the interaction between neutrophils and macrophages during the early phase of the innate immune response to Mtb infection (52) as well as in the control of infectious load and infection-induced changes in lipid metabolism (53), respectively.

Most of the 30 H37Rv-specific host inflammatory response genes identified in the present study are upregulated to affect positively the host-response. These genes lie into the categories of receptors and molecules involved in pro-inflammatory macrophage differentiation (54), recruitment and activation of innate and adaptive immune cells (54–68), type 1 interferon and *IFN-γ* response (69, 70, 71), and apoptosis (72). Almost 30% of those genes are found deregulated in the blood of TB patients and have already been reported to be upregulated during Mtb infection, entailing positive effects on macrophage defense (58, 61, 66, 68, 70, 71, 73–81). Interestingly, miR-132-3p, miR-146a-5p, miR-345-5p and miR-939, which each target as many as 3 or 4 of the H37Rv-specific host inflammatory response genes, have been reported to be implicated in TB or other cellular processes that are or might be relevant to TB. Our IPA analysis of the H37Rv-specific host inflammatory response genes and miRNAs allowed us to identify strong links between Mtb virulence and these genes, as well as their associated pathways.

**Figure 5:**
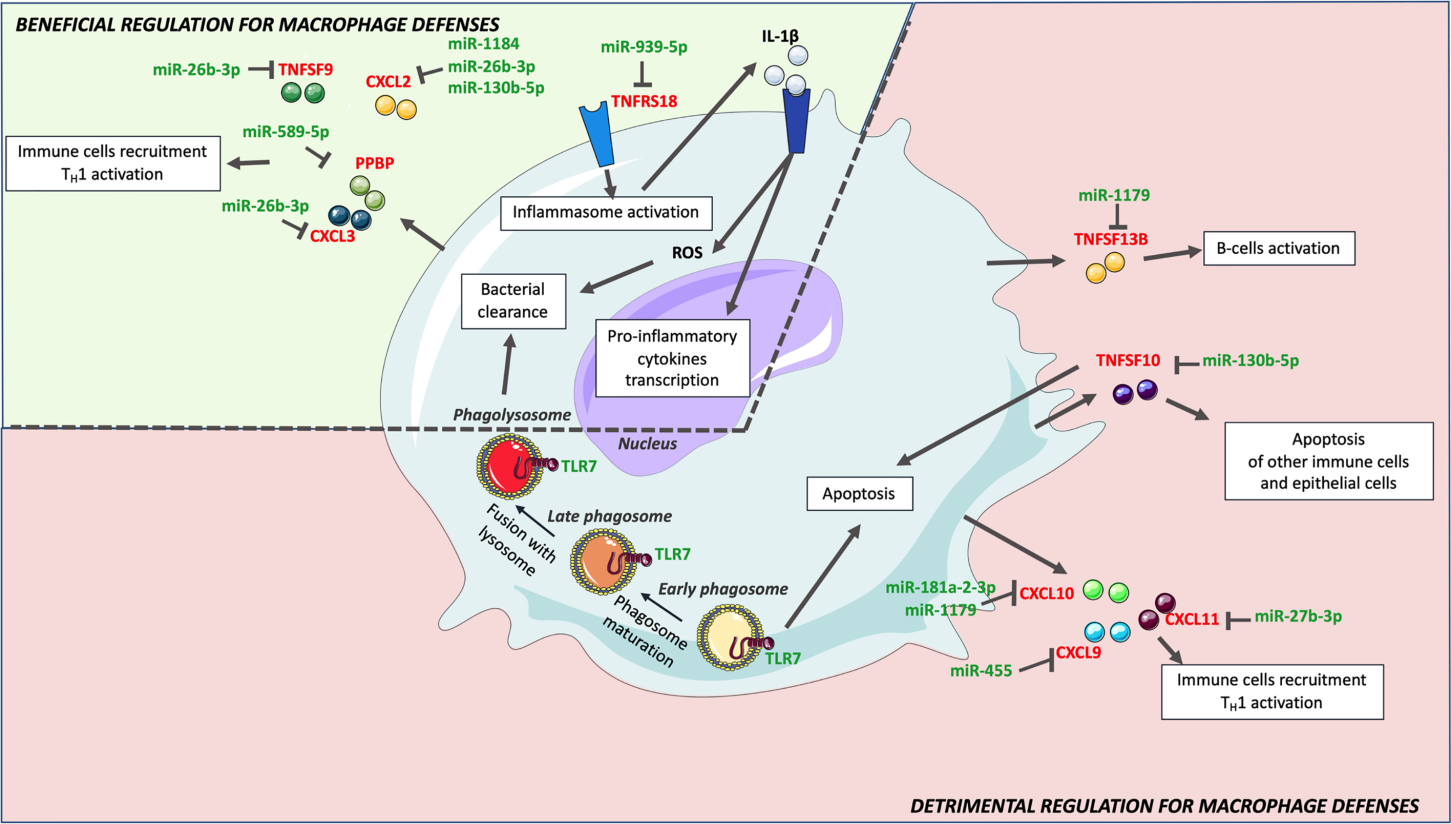
H37R-specific macrophage inflammatory response genes and associated miRNAs. Upon H37Rv infection, macrophages upregulated *CXCL2, CXCL3, IL24*, *PPBP, TNFRSF18* and *TNSF9*, which have a beneficial effect on macrophages defense against Mtb. Downregulation of miR-19b-1-5p, miR-26b-3p, miR-130b-5p, miR-939-5p, miR-589-5p and miR-1184 might however limit the expression of these genes. Although H37Rv led to upregulation of *CCL8, CCL17, CXCL9*, *CXCL10, CXCL11, TNFSF10* and *TNFSF13B*, their expression levels were significantly lower than that induced by *Mm*-infection. Hence, H37Rv mediated lower expression of these genes might rather be detrimental for macrophage defense. Their expression might be limited by miR-27b-3p, miR-130b-5p, miR-181a-2-3p, miR-425p, miR-455 and miR-1179.

Focusing on genes that might benefit or alter macrophages defenses, and pathways in which genes are most targeted by identified miRNAs, we then tried to understand how H37Rv circumvents the macrophage inflammatory response (**Figure 5**). Among the strongest H37Rv-specific host genes/miRNAs displaying inverse expression levels, *CXCL2* is over-expressed following H37Rv infection, and targeted by 3 miRNAs (miR-1184, miR-26b-3p and miR-130b-5p). It has been shown that blocking CXCL2 expression significantly reduces Mtb-induced, but not *M. bovis* BCG-induced, IL-1β secretion through the NLRP3-dependent inflammasome (76, 82), implying that its regulation is related to Mtb virulence. The pro-inflammatory cytokine IL-1β is essential for Mtb control, responsible for activation, for classical inflammatory polarization of macrophages, and takes part in many defense mechanisms against Mtb including ROS production (83, 84). Sustained upregulation of IL-1β leads to detrimental effects of exacerbated inflammation and is associated with TB severity and immunopathology (85–87). The downregulation of miR-1184, miR-26b-3p and miR-130b-5p expressions by H37Rv, might favor CXCL2 upregulation and the subsequent IL-1β production. In addition, the expression level of *TNFRS18*, which is expressed on macrophages, and functions as an inflammatory enhancer by promoting the activation of the NLRP3 inflammasome (88), is inversely correlated with that of miR-939-3p. Interestingly, miR-939-3p directly inhibits iNOS in mouse macrophages (89). iNOS exerts a strong bactericidal activity against Mtb, while preventing an excess of inflammation by inhibiting NLRP3-dependent IL-1β production and the NF-κB pathway (90–92). Moreover, miR-26b-3p, which is thought to participate in NLRP3-dependent IL-1β production through regulation of CXCL2, has been shown to participate in the inflammatory response of LPS-stimulated alveolar macrophages by modulating the NF-κB pathway by regulating PTEN (83). Finally, miR-939-3p and miR-26b-3p, found downregulated in H37Rv-infected macrophages, suggests that NF-κB plays an important role in H37Rv virulence probably through a dialog involving the NLRP3 inflammasome (84, 85). As for many regulatory mechanisms involved in inflammation, these H37Rv-specific gene/miRNA interactions controlling IL-1β might benefit both the macrophage and H37Rv, and highlight the crucial implication of IL-1β-related pathways and the control of pro-inflammatory activation in the virulence of Mtb.

Durable Mtb survival is only possible if their host-cells do not trigger cell death. In the early infection, apoptosis has been shown to be detrimental for Mtb to establish a long-term infection (86). *TNFSF10*, previously reported to be strongly induced by Mtb infection (87), prevents alveolar epithelial cell-mediated wound repair by inducing their apoptosis (93). These data suggest that *TNFSF10* could promote fibrosis of the external layers of the granuloma and favor both the containment and the protection of the bacteria. *TNFSF10* is putatively targeted by miR-130b-5p, which is downregulated in H37Rv-infected macrophages in the present study. In addition to the fact that this miRNA has already been shown to be downregulated in Mtb–infected primary human macrophages (94), P Ahluwalia et al. propose that miR-130b is involved in M1/M2 macrophages plasticity and Mtb survival (95). The expression of *TLR7*, an endosomal receptor expressed by macrophages to detect microbes single stranded RNA (96) and trigger apoptosis (97), is not modulated by *M. marinum* but significantly downregulated by H37Rv, which could also be beneficial for Mtb adaptation to the intracellular life survival process. Moreover, predicted miRNAs associated to the regulation of H37Rv-specific host inflammatory response genes were also involved in the regulation of apoptosis. miR-27b-3p, which expression is decreased upon H37Rv infection, favors apoptosis and decreases bacterial burden in Mtb-infected mouse macrophages (98). MiR-193a-3p and miR-1184, which expressions are decreased upon H37Rv infection, and both putatively target *CXCL2*, are also involved in apoptosis. miR-193a-3p upregulation triggers apoptosis through the direct repression of Mcl-1 expression (99), while miR-1184 promotes apoptosis *via* downregulation of its target CSNK2A1 (100). Reduced expression levels of *TNFSF10, TLR7*, miR-27b-3p, and miR-1184, in H37Rv-infected macrophages as compared with *M. marinum -*infected cells are concordant with apoptosis being reduced in alveolar macrophages infected with the virulent H37Rv strain as compared with attenuated strains such as *M. bovis* BCG and *M. kansasii* (101). The specific inverse regulation of these genes and miRNAs induced by H37Rv might inhibit apoptosis and enhance Mtb virulence.

Another factor that promotes Mtb long-term infection as compared to other pathogens, is the delay between the activation of innate immunity and the recruitment and activation of adaptive immunity (102). The *CXCL9, CXCL10, CXCL11/CXCR3* axis and *TNFSF13B* play a role in both these recruitment and activation processes, responsible for attracting Th1 cells, cytotoxic lymphocytes and natural killer cells to the site of the infection, and related to B-cell activation, respectively (65). Although they were upregulated in H37Rv-infected macrophages, this upregulation was lower than that induced by *M. marinum*, suggesting that they are related to Mtb virulence. The fact that three of these genes are among the 6 most miRNA-targeted genes (i.e. *CXCL9, CXCL11* and *TNFRSF13B*) emphasizes the importance of their role and might explain their lowered expression levels. Upregulation of miR-30a-5p and miR-30d-5p might lower the expression of *CXCL11* and *TNFSF13B*, and upregulation of miR-132-3p that of *CXCL9.* Hence, modulating the expression of those genes and miRNAs might delay the adaptive immunity response and generate permissive conditions for Mtb survival (102). Additionally, miR-30a-5p upregulation is deleterious for Mtb-infected THP-1 cells, as it directly targets MyD88 (103), which is necessary for the establishment of a proper adaptive immune response in many pathologies (104–106). Furthermore, H37Rv-specific modulation of *CCL8, CCL21* and *CXCL2* might also contribute to impairing adaptive immunity. CCL8 is involved in T-cell activation (107–109) and CCL21 is a pro-inflammatory cytokine responsible for T-cells attraction to the lungs (110). Hence, as compared with *M. marinum* infection, lowered expression levels of *CXCL9, CXCL11, TNFSF13B* and *CCL8* and *CCL21*, as well as upregulation of *CXCL2* and miR-30a-5p, might enable H37Rv to expand its population before the burst of adaptive immunity. Consistently with our findings, Goenka et al. (111) also observed lower expression of *CXCL9, CXCL10, CXCL11* and higher expression of *CXCL2* in infant alveolar macrophage than in adult counterparts. With infant alveolar macrophages being more permissive to Mtb infection than adult macrophages (111), this is further evidence that *CXCL2, CXCL9, CXCL10* and *CXCL11* are likely involved in Mtb virulence.

## Conclusion

The fragile balance between beneficial and harmful induction of inflammation might confer to genes and miRNAs paradoxical effects in macrophage defense and Mtb virulence. *CXCL2* induction might both favor IL-1β-mediated protective immunity and impair the recruitment of adaptive immunity. In the broad spectrum of macrophage polarization and corresponding inflammatory responses, Mtb intracellular-linked virulence might depend mostly on: i) the inhibition of IL-1β-dependent pro-inflammatory polarization, ii) the repression of apoptosis, as well as iii) the delay of the adaptive immunity recruitment and activation (**Figure 5**). In this context, macrophage antimicrobial defenses remain low and, once activated, the adaptive immunity maintains this permissive environment.

These miRNA and gene signatures could be the hallmark of the host inflammatory response contribution to Mtb virulence and associated pathways could lead to new host directed therapies.

## Data Availability Statement

The datasets presented in this study can be found in online repositories. The names of the repository/repositories and accession number(s) can be found in the article/**Supplementary Material**.

## Author Contributions

ID-R, FA, and CR supervised the work and designed the experiments. Experimental work was performed by PB, FS, SS, PL, KK, NC and SL. PB, FA, CR, and ID-R analyzed and interpreted the data, and drafted the manuscript. All authors contributed to the article and approved the submitted version.

## Funding

This research was initiated by Sanofi, funded by INSERM (Institut National de la Santé et Recherche Médicale), the University of Montpellier and EVOTEC ID, Lyon. PB received a fellowship from Association Nationale Recherche Technologie (ANRT, convention Cifre N°2017/1678).

## Conflict of Interest

Authors PB, FS, SS, PL, KK, NC, SL and CR were employed by the company Evotec ID.

The remaining authors declare that the research was conducted in the absence of any commercial or financial relationships that could be construed as a potential conflict of interest.​

The authors declare that this study received funding from Evotec ID. The funder had the following involvement with the study: sharing H37rv related protocols, managing training and BSL3 space relative to this study, interpreting data and participating to project meetings.

## References

[1] World Health Organization. Global Tuberculosis Report 2019. World Health Organization (2019). Available at: https://apps.who.int/iris/handle/10665/329368

[2] Diseases TLI. Tuberculosis and Malaria in the Age of COVID-19. Lancet Infect Dis (2021) 21:1. 10.1016/S1473-3099(20)30946-4 33357386 PMC7758173

[3] WHO. Global Tuberculosis Report (2019). Available at: https://www.who.int/tb/publications/global_report/en/ (Accessed 2 July 2020).

[4] Hogan AB Jewell BL Sherrard-Smith E Vesga JF Watson OJ Whittaker C. Potential Impact of the COVID-19 Pandemic on HIV, Tuberculosis, and Malaria in Low-Income and Middle-Income Countries: A Modelling Study. Lancet Global Health (2020) 8:e1132–41. 10.1016/S2214-109X(20)30288-6 PMC7357988 32673577

[5] Bibbins-Domingo K Grossman DC Curry SJ Bauman L Davidson KW Epling JW. Screening for Latent Tuberculosis Infection in Adults: US Preventive Services Task Force Recommendation Statement. JAMA (2016) 316:962–9. 10.1001/jama.2016.11046 27599331

[6] Young D Hussell T Dougan G. Chronic Bacterial Infections: Living With Unwanted Guests. Nat Immunol (2002) 3:1026–32. 10.1038/ni1102-1026 12407411

[7] Flynn JL Chan J Lin PL. Macrophages and Control of Granulomatous Inflammation in Tuberculosis. Mucosal Immunol (2011) 4:271–8. 10.1038/mi.2011.14 PMC3311958 21430653

[8] Schlossberg D. Acute Tuberculosis. Infect Dis Clinics North America (2010) 24:139–46. 10.1016/j.idc.2009.10.009 20171549

[9] Huynh KK Joshi SA Brown EJ. A Delicate Dance: Host Response to Mycobacteria. Curr Opin Immunol (2011) 23:464–72. 10.1016/j.coi.2011.06.002 21726990

[10] Paige C Bishai WR. Penitentiary or Penthouse Condo: The Tuberculous Granuloma From the Microbe’s Point of View. Cell Microbiol (2010) 12:301–9. 10.1111/j.1462-5822.2009.01424.x 20039878

[11] Rubin EJ. The Granuloma in Tuberculosis — Friend or Foe? New Engl J Med (2009) 360:2471–3. 10.1056/NEJMcibr0902539 19494225

[12] Russell DG VanderVen BC Lee W Abramovitch RB Kim M Homolka S. Mycobacterium Tuberculosis Wears What It Eats. Cell Host Microbe (2010) 8:68–76. 10.1016/j.chom.2010.06.002 20638643 PMC2929656

[13] Russell DG Barry CE Flynn JL. Tuberculosis: What We Don’t Know Can, and Does, Hurt Us. Science (2010) 328:852–6. 10.1126/science.1184784 PMC2872107 20466922

[14] Benoit M Desnues B Mege J-L. Macrophage Polarization in Bacterial Infections. J Immunol (2008) 181:3733–9. 10.4049/jimmunol.181.6.3733 18768823

[15] Hernández-Pando R Orozcoe H Sampieri A Pavón L Velasquillo C Larriva-Sahd J. Correlation Between the Kinetics of Th1, Th2 Cells and Pathology in a Murine Model of Experimental Pulmonary Tuberculosis. Immunology (1996) 89:26–33. 8911136 PMC1456655

[16] Lugo-Villarino G Hudrisier D Benard A Neyrolles O. Emerging Trends in the Formation and Function of Tuberculosis Granulomas. Front Immunol (2013) 3:405. 10.3389/fimmu.2012.00405 23308074 PMC3538282

[17] Méndez-Samperio P. Expression and Regulation of Chemokines in Mycobacterial Infection. J Infect (2008) 57:374–84. 10.1016/j.jinf.2008.08.010 18838171

[18] Philips JA Ernst JD. Tuberculosis Pathogenesis and Immunity. Annu Rev Pathol Mech Dis (2012) 7:353–84. 10.1146/annurev-pathol-011811-132458 22054143

[19] Low Penetrance, Broad Resistance, and Favorable Outcome of Interleukin 12 Receptor β1 Deficiency. (Accessed 2 July 2020). Rockefeller University Press.

[20] Impairment of Mycobacterial Immunity in Human Interleukin-12 Receptor Deficiency. (Accessed 2 July 2020). 10.1126/science.280.5368.1432 9603732

[21] Wu K Dong D Fang H Levillain F Jin W Mei J. An Interferon-Related Signature in the Transcriptional Core Response of Human Macrophages to Mycobacterium Tuberculosis Infection. PloS One (2012) 7:e38367. 10.1371/journal.pone.0038367 22675550 PMC3366933

[22] Jenner RG Young RA. Insights Into Host Responses Against Pathogens From Transcriptional Profiling. Nat Rev Microbiol (2005) 3:281–94. 10.1038/nrmicro1126 15806094

[23] Eulalio A Schulte L Vogel J. The Mammalian MicroRNA Response to Bacterial Infections. RNA Biol (2012) 9:742–50. 10.4161/rna.20018 22664920

[24] Wei Y Schober A. MicroRNA Regulation of Macrophages in Human Pathologies. Cell Mol Life Sci (2016) 73:3473–95. 10.1007/s00018-016-2254-6 PMC11108364 27137182

[25] Xiao C Rajewsky K. MicroRNA Control in the Immune System: Basic Principles. Cell (2009) 136:26–36. 10.1016/j.cell.2008.12.027 19135886

[26] Forster SC Tate MD Hertzog PJ. MicroRNA as Type I Interferon-Regulated Transcripts and Modulators of the Innate Immune Response. Front Immunol (2015) 6:334. 10.3389/fimmu.2015.00334 26217335 PMC4495342

[27] Gantier MP Sadler AJ Williams BRG. Fine-Tuning of the Innate Immune Response by MicroRNAs. Immunol Cell Biol (2007) 85:458–62. 10.1038/sj.icb.7100091 17621315

[28] Rajaram MVS Ni B Dodd CE Schlesinger LS. Macrophage Immunoregulatory Pathways in Tuberculosis. Semin Immunol (2014) 26:471–85. 10.1016/j.smim.2014.09.010 PMC4314327 25453226

[29] Sabir N Hussain T Shah SZA Peramo A Zhao D Zhou X. miRNAs in Tuberculosis: New Avenues for Diagnosis and Host-Directed Therapy. Front Microbiol (2018) 9:602. 10.3389/fmicb.2018.00602 29651283 PMC5885483

[30] Behrouzi A Alimohammadi M Nafari AH Yousefi MH Riazi Rad F Vaziri F. The Role of Host Mirnas on Mycobacterium Tuberculosis. ExRNA (2019) 1:40. 10.1186/s41544-019-0040-y

[31] Kim JK Kim TS Basu J Jo E-K. MicroRNA in Innate Immunity and Autophagy During Mycobacterial Infection. Cell Microbiol (2017) 19:e12687. 10.1111/cmi.12687 27794209

[32] Kaufmann SH. How Can Immunology Contribute to the Control of Tuberculosis? Nat Rev Immunol (2001) 1:20–30. 10.1038/35095558 11905811

[33] Clemens DL. Characterization of the Mycobacterium Tuberculosis Phagosome. Trends Microbiol (1996) 4:113–8. 10.1016/0966-842X(96)81528-9 8868090

[34] Bottai D Di Luca M Majlessi L Frigui W Simeone R Sayes F. Disruption of the ESX-5 System of Mycobacterium Tuberculosis Causes Loss of PPE Protein Secretion, Reduction of Cell Wall Integrity and Strong Attenuation. Mol Microbiol (2012) 83:1195–209. 10.1111/j.1365-2958.2012.08001.x 22340629

[35] Jamwal SV Mehrotra P Singh A Siddiqui Z Basu A Rao KVS. Mycobacterial Escape From Macrophage Phagosomes to the Cytoplasm Represents an Alternate Adaptation Mechanism. Sci Rep (2016) 6:23089. 10.1038/srep23089 26980157 PMC4793295

[36] Ehrt S Schnappinger D. Mycobacterial Survival Strategies in the Phagosome: Defense Against Host Stresses. Cell Microbiol (2009) 11:1170–8. 10.1111/j.1462-5822.2009.01335.x PMC3170014 19438516

[37] Simeone R Bobard A Lippmann J Bitter W Majlessi L Brosch R. Phagosomal Rupture by Mycobacterium Tuberculosis Results in Toxicity and Host Cell Death. PloS Pathog (2012) 8:e1002507. 10.1371/journal.ppat.1002507 22319448 PMC3271072

[38] Smith J Manoranjan J Pan M Bohsali A Xu J Liu J. Evidence for Pore Formation in Host Cell Membranes by ESX-1-Secreted ESAT-6 and Its Role in Mycobacterium Marinum Escape From the Vacuole. Infection Immun (2008) 76:5478–87. 10.1128/IAI.00614-08 PMC2583575 18852239

[39] Conrad WH Osman MM Shanahan JK Chu F Takaki KK Cameron J. Mycobacterial ESX-1 Secretion System Mediates Host Cell Lysis Through Bacterium Contact-Dependent Gross Membrane Disruptions. Proc Natl Acad Sci USA (2017) 114:1371–6. 10.1073/pnas.1620133114 PMC5307465 28119503

[40] Linell F Norden A. Mycobacterium Balnei. A New Acid-Fast Bacillus Occurring in Swimming Pools and Capable of Producing Skin Lesions in Humans. Acta Tuberculosea Scandinavica (1954) 33:1–84. 13188762

[41] Gröschel MI Sayes F Shin SJ Frigui W Pawlik A Orgeur M. Recombinant BCG Expressing ESX-1 of Mycobacterium Marinum Combines Low Virulence With Cytosolic Immune Signaling and Improved TB Protection. Cell Rep (2017) 18:2752–65. 10.1016/j.celrep.2017.02.057 28297677

[42] Sato E Imafuku S Ishii K Itoh R Chou B Soejima T. Vitamin D-Dependent Cathelicidin Inhibits Mycobacterium Marinum Infection in Human Monocytic Cells. J Dermatol Sci (2013) 70:166–72. 10.1016/j.jdermsci.2013.01.011 23452544

[43] Coscolla M Gagneux S. Consequences of Genomic Diversity in Mycobacterium Tuberculosis. Semin Immunol (2014) 26:431–44. 10.1016/j.smim.2014.09.012 PMC4314449 25453224

[44] Gan H Lee J Ren F Chen M Kornfeld H Remold HG. Mycobacterium Tuberculosis Blocks Annexin-1 Crosslinking and Thus Apoptotic Envelope Completion on Infected Cells to Maintain Virulence. Nat Immunol (2008) 9:1189–97. 10.1038/ni.1654 PMC5351782 18794848

[45] Sohn H Lee K-S Kim S-Y Shin D-M Shin S-J Jo E-K. Induction of Cell Death in Human Macrophages by a Highly Virulent Korean Isolate of Mycobacterium Tuberculosis and the Virulent Strain H37Rv. Scandinavian J Immunol (2009) 69:43–50. 10.1111/j.1365-3083.2008.02188.x 19140876

[46] Mariotti S Teloni R Iona E Fattorini L Giannoni F Romagnoli G. Mycobacterium Tuberculosis Subverts the Differentiation of Human Monocytes Into Dendritic Cells. Eur J Immunol (2002) 32:3050–8. 10.1002/1521-4141(200211)32:11<3050::AID-IMMU3050>3.0.CO;2-K 12385024

[47] Madhvi A Mishra H Leisching G Mahlobo P Baker B. Comparison of Human Monocyte Derived Macrophages and THP1-Like Macrophages as *In Vitro* Models for M. Tuberculosis Infection. Comp Immunol Microbiol Infect Dis (2019) 67:101355. 10.1016/j.cimid.2019.101355 31586851

[48] Metsalu T Vilo J. Clustvis: A Web Tool for Visualizing Clustering of Multivariate Data Using Principal Component Analysis and Heatmap. Nucleic Acids Res (2015) 43:W566–70. 10.1093/nar/gkv468 PMC4489295 25969447

[49] Lex A Gehlenborg N Strobelt H Vuillemot R Pfister H. Upset: Visualization of Intersecting Sets. IEEE Trans Vis Comput Graph (2014) 20:1983–92. 10.1109/TVCG.2014.2346248 PMC4720993 26356912

[50] Dweep H Gretz N. Mirwalk2.0: A Comprehensive Atlas of MicroRNA-Target Interactions. Nat Methods (2015) 12:697. 10.1038/nmeth.3485 26226356

[51] Chen EY Tan CM Kou Y Duan Q Wang Z Meirelles GV. Enrichr: Interactive and Collaborative HTML5 Gene List Enrichment Analysis Tool. BMC Bioinf (2013) 14:128. 10.1186/1471-2105-14-128 PMC3637064 23586463

[52] Braian C Hogea V Stendahl O. Mycobacterium Tuberculosis-Induced Neutrophil Extracellular Traps Activate Human Macrophages. J Innate Immun (2013) 5:591–602. 10.1159/000348676 23635526 PMC6741595

[53] Johansen MD Hortle E Kasparian JA Romero A Novoa B Figueras A. Analysis of Mycobacterial Infection-Induced Changes to Host Lipid Metabolism in a Zebrafish Infection Model Reveals a Conserved Role for LDLR in Infection Susceptibility. Fish Shellfish Immunol (2018) 83:238–42. 10.1016/j.fsi.2018.09.037 30219383

[54] Xuan W Qu Q Zheng B Xiong S Fan G-H. The Chemotaxis of M1 and M2 Macrophages Is Regulated by Different Chemokines. J Leukocyte Biol (2015) 97:61–9. 10.1189/jlb.1A0314-170R 25359998

[55] Gonçalves AS Appelberg R. The Involvement of the Chemokine Receptor CXCR2 in Neutrophil Recruitment in LPS-Induced Inflammation and in Mycobacterium Avium Infection. Scand J Immunol (2002) 55:585–91. 10.1046/j.1365-3083.2002.01097.x 12028561

[56] Schenk BI Petersen F Flad H-D Brandt E. Platelet-Derived Chemokines CXC Chemokine Ligand (CXCL)7, Connective Tissue-Activating Peptide III, and CXCL4 Differentially Affect and Cross-Regulate Neutrophil Adhesion and Transendothelial Migration. J Immunol (2002) 169:2602–10. 10.4049/jimmunol.169.5.2602 12193731

[57] González-Cortés C Diez-Tascón C Guerra-Laso JM González-Cocaño MC Rivero-Lezcano OM. Non-Chemotactic Influence of CXCL7 on Human Phagocytes. Modulation of Antimicrobial Activity Against L. Pneumophila. Immunobiology (2012) 217:394–401. 10.1016/j.imbio.2011.10.015 22101183

[58] Boro M Singh V Balaji KN. Mycobacterium Tuberculosis-Triggered Hippo Pathway Orchestrates CXCL1/2 Expression to Modulate Host Immune Responses. Sci Rep (2016) 6:37695. 10.1038/srep37695 27883091 PMC5121601

[59] Dorhoi A Iannaccone M Farinacci M Faé KC Schreiber J Moura-Alves P. MicroRNA-223 Controls Susceptibility to Tuberculosis by Regulating Lung Neutrophil Recruitment. J Clin Invest (2013) 123:4836–48. 10.1172/JCI67604 PMC3809781 24084739

[60] Tokunaga R Zhang W Naseem M Puccini A Berger MD Soni S. CXCL9, CXCL10, CXCL11/CXCR3 Axis for Immune Activation - A Target for Novel Cancer Therapy. Cancer Treat Rev (2018) 63:40–7. 10.1016/j.ctrv.2017.11.007 PMC5801162 29207310

[61] Saukkonen JJ Bazydlo B Thomas M Strieter RM Keane J Kornfeld H. β-Chemokines are Induced by Mycobacterium Tuberculosis and Inhibit Its Growth. Infect Immun (2002) 70:1684–93. 10.1128/IAI.70.4.1684-1693.2002 PMC127823 11895930

[62] Sakala IG Eickhoff CS Blazevic A Moheno P Silver RF Hoft DF. Dipterinyl Calcium Pentahydrate Inhibits Intracellular Mycobacterial Growth in Human Monocytes *Via* the C-C Chemokine MIP-1β and Nitric Oxide. Infect Immun (2013) 81:1974–83. 10.1128/IAI.01393-12 PMC3676014 23509148

[63] Hasan Z Cliff JM Dockrell HM Jamil B Irfan M Ashraf M. CCL2 Responses to Mycobacterium Tuberculosis Are Associated With Disease Severity in Tuberculosis. PloS One (2009) 4(12):e8459. 10.1371/journal.pone.0008459 20041183 PMC2793516

[64] Ben-Baruch A Xu L Young PR Bengali K Oppenheim JJ Wang JM. Monocyte Chemotactic Protein-3 (MCP3) Interacts With Multiple Leukocyte Receptors. C-C CKR1, A Receptor for Macrophage Inflammatory Protein-1 Alpha/Rantes, Is Also a Functional Receptor for MCP3. J Biol Chem (1995) 270:22123–8. 10.1074/jbc.270.38.22123 7545673

[65] Ley K Pramod AB Croft M Ravichandran KS Ting JP. How Mouse Macrophages Sense What Is Going On. Front Immunol (2016) 7:204. 10.3389/fimmu.2016.00204 27313577 PMC4890338

[66] Martínez Gómez JM Koh VHQ Yan B Lin W Ang MLT Rahim SZZ. Role of the CD137 Ligand (CD137L) Signaling Pathway During Mycobacterium Tuberculosis Infection. Immunobiology (2014) 219:78–86. 10.1016/j.imbio.2013.08.009 24091276

[67] Gordon S Taylor PR. Monocyte and Macrophage Heterogeneity. Nat Rev Immunol (2005) 5:953–64. 10.1038/nri1733 16322748

[68] Slight SR Khader SA. Chemokines Shape the Immune Responses to Tuberculosis. Cytokine Growth Factor Rev (2012) 24:105–13. 10.1016/j.cytogfr.2012.10.002 PMC3582802 23168132

[69] Ottenhoff THM Dass RH Yang N Zhang MM Wong HEE Sahiratmadja E. Genome-Wide Expression Profiling Identifies Type 1 Interferon Response Pathways in Active Tuberculosis. PloS One (2012) 7:e45839. 10.1371/journal.pone.0045839 23029268 PMC3448682

[70] Wu B Huang C Kato-Maeda M Hopewell PC Daley CL Krensky AM. IL-24 Modulates IFN-γ Expression in Patients With Tuberculosis. Immunol Lett (2008) 117:57–62. 10.1016/j.imlet.2007.11.018 18199488 PMC2679252

[71] Fernández Do Porto DA Jurado JO Pasquinelli V Alvarez IB Aspera RH Musella RM. CD137 Differentially Regulates Innate and Adaptive Immunity Against Mycobacterium Tuberculosis. Immunol Cell Biol (2012) 90:449–56. 10.1038/icb.2011.63 PMC3330265 21747409

[72] Walczak H. Death Receptor–Ligand Systems in Cancer, Cell Death, and Inflammation. Cold Spring Harb Perspect Biol (2013) 5(5):a008698. 10.1101/cshperspect.a008698 23637280 PMC3632055

[73] Torraca V Cui C Boland R Bebelman J-P van der Sar AM Smit MJ. The CXCR3-CXCL11 Signaling Axis Mediates Macrophage Recruitment and Dissemination of Mycobacterial Infection. Dis Models Mech (2015) 8:253–69. 10.1242/dmm.017756 PMC4348563 25573892

[74] Kipnis A Basaraba R Orme I Cooper A. Role of Chemokine CCL2 in the Protective Response to Early Murine Pulmonary Tuberculosis. Immunology (2003) 109:547–51. 10.1046/j.1365-2567.2003.01680.x PMC1783002 12871221

[75] Kang DD Lin Y Moreno J-R Randall TD Khader SA. Profiling Early Lung Immune Responses in the Mouse Model of Tuberculosis. PloS One (2011) 6:e16161. 10.1371/journal.pone.0016161 21249199 PMC3020951

[76] Boro M Balaji KN. CXCL1 and CXCL2 Regulate NLRP3 Inflammasome Activation *Via* G-Protein-Coupled Receptor CXCR2. J Immunol (2017) 199:1660–71. 10.4049/jimmunol.1700129 28739876

[77] Bloom CI Graham CM Berry MPR Rozakeas F Redford PS Wang Y. Transcriptional Blood Signatures Distinguish Pulmonary Tuberculosis, Pulmonary Sarcoidosis, Pneumonias and Lung Cancers. PloS One (2013) 8:e70630. 10.1371/journal.pone.0070630 23940611 PMC3734176

[78] Kaforou M Wright VJ Oni T French N Anderson ST Bangani N. Detection of Tuberculosis in HIV-Infected and -Uninfected African Adults Using Whole Blood RNA Expression Signatures: A Case-Control Study. PloS Med (2013) 10:e1001538. 10.1371/journal.pmed.1001538 24167453 PMC3805485

[79] Gupta RK Turner CT Venturini C Esmail H Rangaka MX Copas A. Concise Whole Blood Transcriptional Signatures for Incipient Tuberculosis: A Systematic Review and Patient-Level Pooled Meta-Analysis. Lancet Respir Med (2020) 8:395–406. 10.1016/S2213-2600(19)30282-6 31958400 PMC7113839

[80] Berry MPR Graham CM McNab FW Xu Z Bloch SAA Oni T. An Interferon-Inducible Neutrophil-Driven Blood Transcriptional Signature in Human Tuberculosis. Nature (2010) 466:973–7. 10.1038/nature09247 PMC3492754 20725040

[81] Lavalett L Rodriguez H Ortega H Sadee W Schlesinger LS Barrera LF. Alveolar Macrophages From Tuberculosis Patients Display An Altered Inflammatory Gene Expression Profile. Tuberculosis (2017) 107:156–67. 10.1016/j.tube.2017.08.012 29050765

[82] Bourigault M-L Segueni N Rose S Court N Vacher R Vasseur V. Relative Contribution of IL-1α, Il-1β and TNF to the Host Response to Mycobacterium Tuberculosis and Attenuated M. Bovis BCG. Immun Inflammation Dis (2013) 1:47–62. 10.1002/iid3.9 PMC4217540 25400917

[83] Microrna-26b Modulates the NF-κb Pathway in Alveolar Macrophages by Regulating PTEN (Accessed 3 May 2021). 10.4049/jimmunol.1402933 PMC4655123 26503952

[84] Boaru SG Borkham-Kamphorst E Van de Leur E Lehnen E Liedtke C Weiskirchen R. NLRP3 Inflammasome Expression Is Driven by NF-κb in Cultured Hepatocytes. Biochem Biophys Res Commun (2015) 458:700–6. 10.1016/j.bbrc.2015.02.029 25686493

[85] Kinoshita T Imamura R Kushiyama H Suda T. Nlrp3 Mediates Nf-κb Activation and Cytokine Induction in Microbially Induced and Sterile Inflammation. PloS One (2015) 10:e0119179. 10.1371/journal.pone.0119179 25761061 PMC4356585

[86] Dheda K Booth H Huggett JF Johnson MA Zumla A Rook GAW. Lung Remodeling in Pulmonary Tuberculosis. J Infect Dis (2005) 192:1201–10. 10.1086/444545 16136463

[87] Badr MT Häcker G. Gene Expression Profiling Meta-Analysis Reveals Novel Gene Signatures and Pathways Shared Between Tuberculosis and Rheumatoid Arthritis. PloS One (2019) 14:e0213470. 10.1371/journal.pone.0213470 30845171 PMC6405138

[88] Liang S Liu Y Wu Y Ming S Wu M. GITR Expressed on Macrophages Functions as an Inflammatory Amplifier by Promoting NLRP3 Inflammasome Activation. J Immunol (2020) 204:68.11–1.

[89] Li M Wang J Fang Y Gong S Li M Wu M. Microrna-146a Promotes Mycobacterial Survival in Macrophages Through Suppressing Nitric Oxide Production. Sci Rep (2016) 6:23351. 10.1038/srep23351 27025258 PMC4812255

[90] Mishra BB Rathinam VAK Martens GW Martinot AJ Kornfeld H Fitzgerald KA. Nitric Oxide Controls the Immunopathology of Tuberculosis by Inhibiting NLRP3 Inflammasome-Dependent Processing of IL-1β. Nat Immunol (2013) 14:52–60. 10.1038/ni.2474 23160153 PMC3721324

[91] Mishra BB Lovewell RR Olive AJ Zhang G Wang W Eugenin E. Nitric Oxide Prevents a Pathogen-Permissive Granulocytic Inflammation During Tuberculosis. Nat Microbiol (2017) 2:1–11. 10.1038/nmicrobiol.2017.72 PMC5461879 28504669

[92] Braverman J Stanley SA. Nitric Oxide Modulates Macrophage Responses to Mycobacterium Tuberculosis Infection Through Activation of HIF-1α and Repression of NF-κb. J Immunol (2017) 199:1805–16. 10.4049/jimmunol.1700515 PMC5568107 28754681

[93] Akram KM Lomas NJ Spiteri MA Forsyth NR. Club Cells Inhibit Alveolar Epithelial Wound Repair *Via* TRAIL-Dependent Apoptosis. Eur Respir J (2013) 41:683–94. 10.1183/09031936.00213411 22790912

[94] Ni B Rajaram MVS Lafuse WP Landes MB Schlesinger LS. Mycobacterium Tuberculosis Decreases Human Macrophage IFN-γ Responsiveness Through Mir-132 and Mir-26a. J Immunol (2014) 193:4537–47. 10.4049/jimmunol.1400124 25252958

[95] Ahluwalia PK Pandey RK Sehajpal PK Prajapati VK. Perturbed Microrna Expression by Mycobacterium Tuberculosis Promotes Macrophage Polarization Leading to Pro-Survival Foam Cell. Front Immunol (2017) 8:107. 10.3389/fimmu.2017.00107 28228760 PMC5296369

[96] Kim Y-M Brinkmann MM Paquet M-E Ploegh HL. UNC93B1 Delivers Nucleotide-Sensing Toll-Like Receptors to Endolysosomes. Nature (2008) 452:234–8. 10.1038/nature06726 18305481

[97] Yu X Wang Y Zhao W Zhou H Yang W Guan X. Toll-Like Receptor 7 Promotes the Apoptosis of THP-1-Derived Macrophages Through the CHOP-Dependent Pathway. Int J Mol Med (2014) 34:886–93. 10.3892/ijmm.2014.1833 24994112

[98] Liang S Song Z Wu Y Gao Y Gao M Liu F. MicroRNA-27b Modulates Inflammatory Response and Apoptosis During Mycobacterium Tuberculosis Infection. J Immunol (2018) 200:3506–18. 10.4049/jimmunol.1701448 29661829

[99] Kwon J-E Kim B-Y Kwak S-Y Bae I-H Han Y-H. Ionizing Radiation-Inducible MicroRNA Mir-193a-3p Induces Apoptosis by Directly Targeting Mcl-1. Apoptosis (2013) 18:896–909. 10.1007/s10495-013-0841-7 23546867

[100] Chen S Wang Y Xu M Zhang L Su Y Wang B. Mir-1184 Regulates the Proliferation and Apoptosis of Colon Cancer Cells *Via* Targeting CSNK2A1. Mol Cell Probes (2020) 53:101625. 10.1016/j.mcp.2020.101625 32619668

[101] Keane J Remold HG Kornfeld H. Virulent Mycobacterium Tuberculosis Strains Evade Apoptosis of Infected Alveolar Macrophages. J Immunol (2000) 164:2016–20. 10.4049/jimmunol.164.4.2016 10657653

[102] Wolf AJ Desvignes L Linas B Banaiee N Tamura T Takatsu K. Initiation of the Adaptive Immune Response to Mycobacterium Tuberculosis Depends on Antigen Production in the Local Lymph Node, Not the Lungs. J Exp Med (2008) 205:105–15. 10.1084/jem.20071367 PMC2234384 18158321

[103] Wu Y Sun Q Dai L. Immune Regulation of Mir-30 on the Mycobacterium Tuberculosis-Induced TLR/Myd88 Signaling Pathway in THP-1 Cells. Exp Ther Med (2017) 14:3299–303. 10.3892/etm.2017.4872 PMC5585754 28912881

[104] Nance SC Yi A-K Re FC Fitzpatrick EA. Myd88 is Necessary for Neutrophil Recruitment in Hypersensitivity Pneumonitis. J leukocyte Biol (2008) 83:1207. 10.1189/jlb.0607391 18285403 PMC2626771

[105] Babcock AA Toft-Hansen H Owens T. Signaling Through Myd88 Regulates Leukocyte Recruitment After Brain Injury. J Immunol (2008) 181:6481–90. 10.4049/jimmunol.181.9.6481 18941239

[106] Loures FV Pina A Felonato M Feriotti C de Araújo EF Calich VLG. Myd88 Signaling Is Required for Efficient Innate and Adaptive Immune Responses to Paracoccidioides Brasiliensis Infection. Infection Immun (2011) 79:2470. 10.1128/IAI.00375-10 PMC3125857 21422180

[107] Liu H Liu Z Chen J Chen L He X Zheng R. Induction of CCL8/MCP-2 by Mycobacteria Through the Activation of TLR2/PI3K/Akt Signaling Pathway. PloS One (2013) 8(2):e56815. 10.1371/journal.pone.0056815 23418602 PMC3572057

[108] Uguccioni M D’Apuzzo M Loetscher M Dewald B Baggiolini M. Actions of the Chemotactic Cytokines MCP-1, MCP-2, MCP-3, RANTES, MIP-1 Alpha and MIP-1 Beta on Human Monocytes. Eur J Immunol (1995) 25:64–8. 10.1002/eji.1830250113 7531149

[109] Asano K Takahashi N Ushiki M Monya M Aihara F Kuboki E. Intestinal CD169 + Macrophages Initiate Mucosal Inflammation by Secreting CCL8 That Recruits Inflammatory Monocytes. Nat Commun (2015) 6:7802. 10.1038/ncomms8802 26193821 PMC4518321

[110] Khader SA Rangel-Moreno J Fountain JJ Martino CA Reiley WW Pearl JE. In a Murine Tuberculosis Model, the Absence of Homeostatic Chemokines Delay Granuloma Formation and Protective Immunity. J Immunol (2009) 183:8004–14. 10.4049/jimmunol.0901937 PMC2799945 19933855

[111] Goenka A Prise IE Connolly E Fernandez-Soto P Morgan D Cavet JS. Infant Alveolar Macrophages Are Unable to Effectively Contain Mycobacterium Tuberculosis. Front Immunol (2020) 11:486. 10.3389/fimmu.2020.00486 32265931 PMC7107672

