# Supplementary material for: Integrative Analysis of Human Macrophage Inflammatory Response Related to Mycobacterium tuberculosis Virulence
Source: Front Immunol. 2021 Jun 28;12:668060. doi: 10.3389/fimmu.2021.668060 (PMC8284339; doi:10.3389/fimmu.2021.668060)
Supplement: Supplementary file 1 [file DataSheet_1.pdf]

Integrative analysis of human macrophage inflammatory response related to *Mycobacterium tuberculosis* virulence

Pauline Bade [1,2], Fabrizio Simonetti[2], Stephanie Sans[2], Patricia Laboudie[2], Khadija Kissane[2], Nicolas Chappat[2], Sophie Lagrange[2], Florence Apparailly[1], Christine Roubert[2, #] and Isabelle Duroux-Richard[1, #, *]

Supplementary Material

1 Supplementary Figure

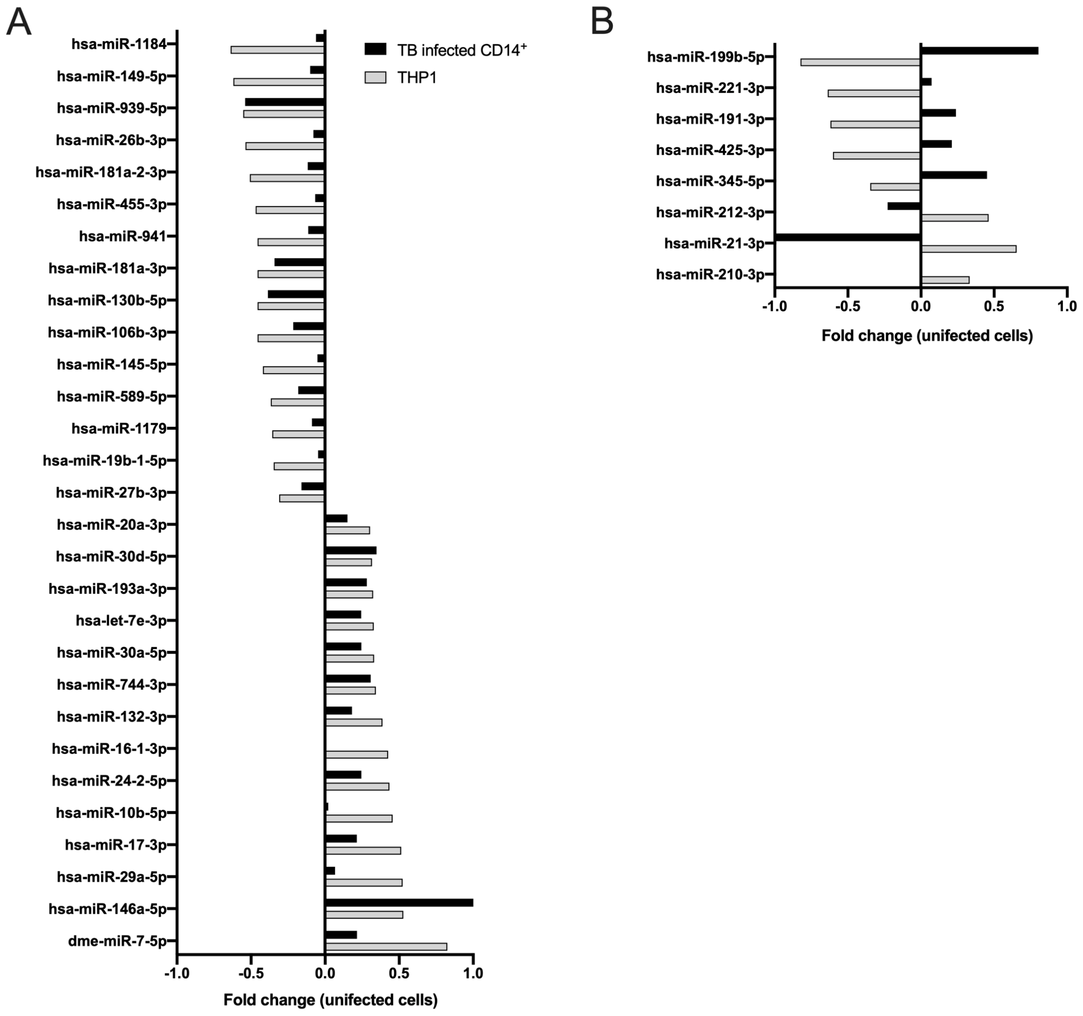

Supplementary Figure 1: Infection with tuberculosis led to a specific miRNA signature. Using a miRNome published dataset (GSE70425) of CD14+ monocytes isolated from TB blood patients, we compared the expression levels of the 37 H37Rv-related miRNAs between the Mtb-infected THP-1

cell line and primary CD14^{+} cells. Graphs represent miRNA expression fold changes in either THP-1 compared to uninfected cells (THP-1, grey bare) or CD14^{+} blood monocytes from TB patients compared to healthy donors (n=7), (CD14^{+}, black bare). **A**) and **B**) miRNAs with the same or different expression levels between THP-1 and CD14^{+}, respectively.
